# Pride and Social Status

**DOI:** 10.3389/fpsyg.2018.01979

**Published:** 2018-10-25

**Authors:** Henrietta Bolló, Beáta Bőthe, István Tóth-Király, Gábor Orosz

**Affiliations:** ^1^Doctoral School of Psychology, Eötvös Loránd University, Budapest, Hungary; ^2^Institute of Psychology, University of Eötvös Loránd, Budapest, Hungary; ^3^Institute of Cognitive Neuroscience and Psychology, Research Centre for Natural Sciences, Hungarian Academy of Sciences, Budapest, Hungary; ^4^Department of Psychology, Stanford University, Stanford, CA, United States

**Keywords:** authentic pride, hubristic pride, objective status, status maintenance strategy, subjective status

## Abstract

Pride is a status-related self-conscious emotion. The present study aimed to investigate the nature of status behind pride in four studies with using the two-facet model of pride, status maintenance strategies and with differentiating subjective social status (SSS) and objective social status (OSS). In Studies 1 and 2, we used questionnaire methods with structural equation modeling (SEM) in order to identify the relationship patterns between SSS, OSS, status maintenance strategies and pride. In Studies 3 and 4, we used vignette method and SEM to identify these links. All four studies gave evidence for the SSS → prestige status maintenance strategy → authentic pride relationship pattern. Similarly consistent result was found regarding the dominance status maintenance strategy → hubristic pride link. Depending on the assessment method (questionnaire vs. vignette) and the evaluative frame of reference (self vs. other), OSS was related to either authentic and hubristic pride, only hubristic pride, or neither of them. Based on these results, one thing can be taken for granted: pride is a subjective status-related emotion. However, the present results suggest that it is not necessarily true for OSS.

## Introduction

Pride has a fundamental affective role in status seeking, attaintment, and signaling (Cheng et al., 2010, 2013). The social function of pride is to express high status which is beneficial for both displayers and observers (Martens et al., 2012). Displayers receive deference from others while observers get valuable information about the allocation of resources. Despite pride is a status-related emotion, we have limited knowledge about how this self-conscious emotion is related to the two main forms of social status by considering its subjective and objective aspects. In this research, we aimed to overcome this limitation by investigating these two forms of social status, different status maintenance strategies, as well as the two facets of pride. Many attempts were made to examine the different outcomes and predictors of the two facets of pride (e.g., Tracy and Robins, 2007a,b; Tracy et al., 2010). However, less research focused on the decomposition of its most important element: status.

According to the functionalist view of social status, clear hierarchies are advantageous for the group because the desire for reaching higher status motivates group-oriented behaviors (Griskevicius et al., 2010). In line with the functionalist view, micropolitics theory (Anderson and Cowan, 2014; Anderson and Willer, 2014) proposes that there are two fundamental processes of how an individual’s social status is composed: group members’ evaluations on who deserves higher status and the candidate’s motivation for reaching higher status. This theory defines status as a function of the group members’ evaluations and decisions about who deserves high rank (Bales et al., 1951; Berger et al., 1972). Group members make a consensus on what features are appreciated for high status and they evaluate each member along these qualities. If a higher status candidate possesses most of these characteristics he/she will be approved for higher status by other members of the group. These evaluations of group members are basically subjective and not always in line with the characteristics or behavior of the evaluated individuals (Anderson and Cowan, 2014). According to micropolitics theory individuals are not passive recipients of status but they actively seek and attain current or higher status.

Consequently, group members are motivated to increase their value in the eyes of other group members by emphasizing those qualities which fit the preferred status. As the status evaluation of the group members is subjective, affective components play a key role both in status display and status perception processes. Among the relevant emotions, pride is one of the most important one that facilitates the navigation in the social hierarchy (Steckler and Tracy, 2014).

Based on Steckler and Tracy (2014), affects can be related to social status in three distinct, yet interrelated ways. First, the *experience* of a status related emotion promotes such behaviors which facilitate the navigation in the social hierarchy. According to the “affect as information” hypothesis (Schwarz and Clore, 1983; Clore et al., 2001), emotions have a function to inform individuals about their relative social worth. In other words, by perceiving their own internal states, individuals draw conclusions about their social context. Moreover, based on motivational theories, emotions can directly motivate behaviors to improve social rank. The subjective experience of pride can inform the group member about his/her high social rank and promotes the maintenance of this high status by certain status-maintenance strategies (Cheng et al., 2010). Second, *non-verbal displays* of status-related emotions may help the navigation in a social hierarchy as they represent one’s current social rank or a change in social rank to observers (Tiedens and Fragale, 2003). Communicating status-relevant information helps group members to avoid costly disputes which can appear when individuals’ social rank levels are unknown. Therefore, signaling status may allow group members to quickly know how social interactions should proceed. For example, manifested pride displays are important signals of high social status even in the presence of contradicting contextual information (Cheng et al., 2010; Tracy et al., 2010). Third, closely connected to this approach, emotions influence social navigation when they are *perceived* by others. Recognizing and automatically interpreting status-relevant emotions and their meaning, perceivers are able to adjust their behavior in an adaptive manner. For example, perceiving pride gives information about high social rank so individuals know who to respect, and who has control over resources. The process of displaying and perceiving emotions are almost the same, but it is important to emphasize the different benefits for both displayers and observers. In sum, in evolutionary terms, signaling pride is a cost-effective and peaceful way of status attaintment as it facilitates the communication between group members with different ranks.

Based on evolutionary theory and supported by empirical research, Tracy et al. (2010) established the Two-facet Model of pride. They differentiated authentic and hubristic pride which have evolved to maintain status in different ways (Tracy and Robins, 2007a,b).

*Authentic pride* is experienced when the attribution of success is internal, unstable, and controllable (Lewis, 2007; Tracy et al., 2009). Authentic pride is associated with extraversion, agreeableness, conscientiousness, satisfying social relationships, high self-esteem, prosocial behaviors, achievement-orientation and mental health (Tracy and Robins, 2007a,b; Tracy et al., 2009; Cheng et al., 2010).

*Hubristic pride* is experienced if the attribution of success is external, stable, and uncontrollable (Lewis, 2007; Tracy et al., 2009). In contrast to authentic pride, it is related to more antisocial and aggressive behaviors. It is associated with disagreeableness, neuroticism, lack of conscientiousness, narcissism, problematic relationships, and poor mental health outcomes (Tracy and Robins, 2007a,b; Tracy et al., 2009; Cheng et al., 2010).

Furthermore, a main difference between the two facets of pride is that authentic and hubristic pride have evolved to motivate different status maintenance strategies. More precisely, authentic pride is related to prestige-based status maintenance and hubristic pride is related to dominance-based status maintenance (Cheng et al., 2010, 2013) and both dominance and prestige refer to the attainment of high social rank.

*Dominant strategies* include intimidating subordinates by threatening them with retaining resources and it is positively related to narcissistic self-aggrandizement, aggression, and negatively related to agreeableness (Henrich and Gil-White, 2001; Cheng et al., 2010). The psychological correlates of hubristic pride contribute to dominance-based status maintenance. In other words, the subjective experience of arrogance and superiority, deriving from hubristic pride promotes the individual to be capable of using threatening strategies, related to dominance (Cheng et al., 2010).

However, individuals using *prestige-based status maintenance strategies* are not feared but respected by group members because they possess cultural knowledge and skills and they are open to share these resources (Henrich and Gil-White, 2001). Prestige is negatively related to aggression and neuroticism and positively related to genuine self-esteem, social acceptance, extraversion, conscientiousness, openness, and authentic pride (Cheng et al., 2010, 2013). Authentic pride contributes to prestige-based status maintenance as it mentally predisposes the individual to be capable of using constructive strategies (i.e., confidence, agreeableness, openness, and accomplishment) to be respected by others (Cheng et al., 2010).

Nevertheless the Two-facet model by Tracy and Robins (2004, 2007a,b) is well supported by numerous and multimethod empirical research, an alternative conceptualization has to be taken into account. The Merited Success/Unmerited Display model (M/U model) of pride (Holbrook et al., 2014a,b) questions the construct validity of the Two-facet model considering how the facets of pride are measured by the seven-item Authentic and Hubristic Pride Scale (Tracy and Robins, 2007b). The Two-facet model claims that in the case of authentic pride, success is attributed to personal efforts, but not to ability, so in case of failure, authentic pride will be related to lack of effort. Instead, the M/U model suggests that in the case of authentic pride, success can be attributed to both effort and ability. Meanwhile, authentic pride will not promote attributions of failure to the individual at all because well deserved pride –like authentic pride – is antithetical to failure. Furthermore, the Two-facet model claims that in the case of hubristic pride, success is attributed to personal abilities, but not to effort, so in case of failure, pride will be related to lack of personal capacities. Instead, the M/U model suggests that in the case of hubristic pride success cannot be attributed neither to effort nor to ability. According to the items of the seven-item scale, hubristic pride is displayed when the success is perceived by the individual as unmerited so pride displays are excessive in this sense. Therefore, the M/U model puts more emphasis on the role of attributional and appraisal processes regarding the experience of pride. According to Tracy and Robins (2014), hubristic pride can appear only with the underlying experience and it is impossible to display pride without the related emotions (as in the M/U model).

Thus, there are some contradictions regarding how authentic and hubristic pride is evoked by success. To understand the underlying dynamics behind the two facets, we propose to take a step back. Based on the process model of self-conscious emotions (Tracy and Robins, 2004, 2007a) pride is experienced after complex cognitive evaluations of the eliciting event. These evaluations include causal attributions of emotions (e.g., Weiner, 1985), cognitive appraisal theory (e.g., Lazarus, 1991; Scherer, 2001), and self-evaluative processes (e.g., Higgins, 1987; Carver and Scheier, 1998). As pride is one of the most relevant status-related emotions, investigating the nature of this eliciting event, namely the gained status, may be relevant to understand the dynamics of the two facets.

Although the relationship pattern of pride and status maintenance is well-known and supported by empirical evidence, these studies did not investigate what type of social status is maintained by prestige or dominance. In this research, we focused on the relationship between pride and social status from a new perspective by investigating objective social status (OSS) and subjective social status (SSS) separately. In previous studies, differentiating between the objective and subjective side of social status seemed to be relevant regarding many psychological constructs, such as negative affectivity, pessimism, stress, control over life, active and passive coping (Adler et al., 2000), mental health (Franzini and Fernandez-Esquer, 2006), well-being (Howell and Howell, 2008), depressive symptoms (Hoebel et al., 2017) and the probability for experiencing shame (Lundberg et al., 2009). Based on these previous results, investigating the role of two different types of status regarding a status-relevant emotion might be relevant. Differentiating subjective and objective status can give us a deeper insight into the dynamics of status-related pride. It is important to know what kind of status is relevant to feel authentic pride because it can has several applied implications, for example in a workplace context (Lu and Roto, 2016).

By definition *subjective social status* means the individuals’ own perception of their relative position in the social hierarchy (Jackman and Jackman, 1973; Adler and Stewart, 2007). According to one of the most common assessment of SSS individuals with high status receive respect, admiration from the significant groups and they have large influence in these groups. However, low status members receive no respect, no admiration, and have no influence in these groups (Shaked et al., 2016). The level of SSS can be represented on a “social ladder” where high status individuals take place on the top, while low status individuals are on the bottom of the ladder. In sum, SSS refers to the perceived relative position in important reference groups which is based on perceived respect, admiration and influence.

Contrasting to SSS, *objective social status* consists of measures of such status indicators as education, income, occupation, financial wealth, household goods, type of habitation, and type of car, etc. (Adler and Stewart, 2007). Therefore, perceived objective status is based on possessions, tangible resources and educational background which do not necessarily involve perceived respect, admiration and influence. Although these two types of social status are correlated but have different outcomes (Goldman et al., 2006). We expect that this differentiation can be visible regarding not only health-related outcomes (Cundiff and Matthews, 2017), but regarding such self-conscious, status-related emotions as authentic and hubristic pride.

Centers (1949) emphasized that individuals who were classified as belonging to poorer socioeconomic groups, did not have to think about themselves as inferior to others. In relevant social groups (e.g., family, friends), these individuals may experience admiration or respect as a result of skills or knowledge, leading to higher levels of SSS. In line with this, those with the highest OSS may feel unappreciated and unrespected (low SSS) by others. Moreover, SSS may reflect not only the current social circumstances of an individual but also incorporates with the individual’s past or future prospects (Singh-Manoux et al., 2005). It can explain that someone can have a high SSS without actually high OSS.

In the current research, we hypothesized that OSS and SSS have differentiated effect on the two facets of pride. First, as SSS is based on perceived respect, admiration and influence of the reference groups, we hypothesized that SSS is more strongly related to pride than OSS. Pride is interpreted as the outcome emotion of the group’s subjective evaluation of the given person’s success (Tracy and Robins, 2004), which is mainly based on the feedback of the relevant social groups and less on the objective resources (e.g., level of education, money, different goods). Second, we expected that SSS and OSS are not only directly related to the two facets of pride, but prestige and dominance can play a mediating role as well.

We expected that SSS will be related to authentic pride via prestige for the following reasons: if individuals experience respect, admiration and influence in their relevant social groups they will be able to use prestige-based status maintenance strategies such as sharing cultural resources, like their skills and knowledge which can promote authentic pride (Cheng et al., 2010, 2013). Individuals with high SSS do not have to experience being threatened by losing their position, as the group members confirm their status with expressing respect and admiration and this experience can promote authentic pride. Individuals with high SSS may not have to apply dominant strategies such as threatening others by withholding resources and being aggressive in order to maintain their SSS – which is based on respect and admiration – and this cognition may mentally predispose the individual to experience authentic pride. Furthermore, this relationship pattern can create a positive loop, because authentic pride displays are socially more accepted so they boost social status (Williams and DeSteno, 2009). In line with this authentic pride can become the underlying affective mechanism of prestige-based status maintenance of high SSS. Moreover, if SSS is maintained by dominance, the underlying mechanism of threatening others may mentally predispose the individual to experience hubristic pride.

On the other hand, we expected that OSS can be relevant as well, regarding pride is related to high status, and high status means privileged access to resources. In the material society, these resources can be money, education and social institutions (Kafashan et al., 2014). Furthermore, we expected that OSS will be a more relevant background variable in case of hubristic pride, especially if it is maintained by dominance-based strategies. We expected it is especially true if one has low level of SSS and high level of OSS. It means, that individuals who have abundant resources in terms of high level of education, money, possessions, etc. but not respected or admired by others and have no influence, need to maintain their status in the hierarchy by dominant strategies which mentally predispose the individual to experience that s/he is conceited, stuck-up, namely proud, but in a hubristic way (Cheng et al., 2010, 2013). This can also create a feedback loop, but in this case a negative one, contrasting to the SSS→prestige→authentic pride circle. However, as it was mentioned above, we expected larger effects in the case of SSS than OSS on the two forms of pride. Furthermore, as the two forms of pride correlated in prior studies (Tracy and Robins, 2007b), cross-effects between OSS, SSS, status maintenance strategies and facets of pride can emerge.

We investigated these predictions in four studies. In Study 1, we investigated these predictions with a self-reported online questionnaire. Study 2 was a similar self-reported questionnaire study but with a multidimensional measure of OSS. In Study 3, OSS and SSS were manipulated in a 2 × 2 vignette design and participants were requested to indicate their hypothetical emotions and behaviors in these situations. Study 4 had the same vignette design as Study 3 with only one exception that participants had to evaluate an imagined other person’s feelings and behaviors and not their own.

## Study 1

In Study 1, we investigated how OSS and SSS is associated with status maintenance strategies and pride in a self-reported questionnaire study. SSS was assessed with the McArthur ladder (Adler and Stewart, 2007) which represented where individuals stand in their relevant social groups regarding respect, admiration and influence. OSS was assessed with level of education and monthly income. SEM analysis was carried out to investigate the relationship pattern of SSS, OSS and pride with the mediation of dominance and prestige. The raw data is available on OSF: https://osf.io/ebg8a/ conclusions of this manuscript will be made available by the authors, without undue reservation, to any qualified researcher.

### Method

#### Participants

A total of 552 Hungarian participants were recruited from topic irrelevant social media groups with more than 10,000 members in the present study (488 females), aged between 18 and 76 (*M*_age_ = 30.66 years, *SD*_age_ = 10.35 years). Regarding their level of education, 333 of them had university degree (60.3%), 192 (34.8%) had high school degree, 25 (4.5%) had elementary school degree, and two participants (0.4%) had no elementary degree. Regarding their place of residence, 194 (35.1%) lived in the capital, 70 (12.7%) lived in county towns, 222 (40.2%) lived in towns, and 66 (12.0%) lived in villages. Respondents were also asked about their average monthly income (*M*_Hungarianincome_ = 372 USD as per the Hungarian Central Statistical Office, 2006). The average monthly income for 79 (14.3%) respondents was less than 180 USD, 187 respondents (33.9%) had between 180 and 540 USD, 169 (30.6%) had an average monthly income of 541–904 USD, 86 (15.6%) had 905–1,808 USD monthly income on average, 21 (3.8%) respondents had more than 1809 USD average monthly income and 10 individuals (1.8%) did not indicate their average monthly income.

#### Measures

##### Hubristic and Authentic Pride Scale

This measure (Tracy and Robins, 2007b) consisted of seven authentic items (e.g., accomplished, fulfilled; α = 0.87) and seven hubristic pride items (e.g., stuck-up, conceited; α = 0.84). Respondents had to indicate the extent to which they generally felt using a 5-point scale (1 = Not at all; 5 = Extremely). All translated measures in the present research were translated in Hungarian using the protocol of Beaton et al. (2000). Because it was the first Hungarian adaptation of the scale Confirmatory Factor Analysis was conducted (TLI = 0.969, CFI = 0.978, RMSEA = 0.053). The final scale consisted of five items on both subscales. (We eliminated the items “confident,” “like I have self-worth,” “egotistical,” and “smug” based on factor loadings and face validity.)

##### Dominance and Prestige Scale

This questionnaire (Cheng et al., 2010) consisted of 10 dominance items (e.g., “I am willing to use aggressive tactics to get my way”; α = 0.76) and 12 prestige items (e.g., “My unique talents and abilities are recognized by others”; α = 0.80). Respondents had to indicate their level to which the items described them using a 7-point scale (1 = Not at all; 7 = Very much). Because it was the first Hungarian adaptation of the scale Confirmatory Factor Analysis was conducted (TLI = 0.963, CFI = 0.980, RMSEA = 0.044). The final scale consisted of three items on both subscales. Dominance subscale consisted of the following items: “I dislike giving orders. (reversed item),” “I enjoy having control over others.” and “I enjoy having authority over other people.” Prestige subscale consisted of the following items: “Others seek my advice on a variety of matters.” “I have gained distinction and social prestige among others in the group.” and “I am considered an expert on some matters by others.”

##### MacArthur scale of subjective social status

Subjective social status was measured by a 10-point social ladder (Adler et al., 2000; Ostrove et al., 2000) in which respondents were asked to indicate their position if “1” represented those who are the most disdained in their social groups and “10” represented those who are the most successful, the most admired in the relevant social groups, which can be family, friends, colleagues, etc. According to the original definition of the ladder by Adler et al. (2000) participants were allowed to define their own groups.

##### Objective social status

Objective social status was assessed with typical socioeconomic status indicators such as educational level (1 = less than elementary school degree; 2 = finished elementary school; 3 = ongoing high school; 4 = finished high school; 5 = ongoing higher education; 6 = finished university) and monthly income (1 = between 0 and 50,000 HUF ∼ 0 and 180 USD; 2 = between 50,001 and 150,000 HUF ∼ 181 and 540 USD; 3 = between 150,001 and 250,000 HUF ∼ 541 and 900 USD; 4 = between 250,001 and 500,000 HUF ∼ 901 and 1,800 USD; 5 = above 500,001 HUF ∼ 1,800 USD) with the categories mentioned above.

#### Procedure

This study was performed with an online questionnaire system. First, participants were informed about the goals and the content of the study. They were also assured the anonymity of their answers. The first part of questionnaire contained the Hubristic and Authentic Pride Scale, followed by the Dominance and Prestige Scale. In the second part, demographic questions were asked, including the measures of SSS and OSS. This research was approved by the Research Ethics Committee of Eötvös Loránd University Faculty of Education and Psychology and was carried out in accordance with Declaration of Helsinki. All subjects gave written informed consent.

#### Statistical Analysis

Structural equation modeling (SEM) was implemented to assess the effect of OSS and SSS on prestige, dominance, authentic, and hubristic pride. When assessing the model, multiple goodness of fit indices were taken into account (Bentler, 1990; Browne and Cudeck, 1993; Schermelleh-Engel et al., 2003; Hu and Bentler, 2006; Brown, 2015). The Comparative Fit Index (CFI; good > 0.90), the Tucker-Lewis Index (TLI; good > 0.90) and the root mean square error of approximation (RMSEA; good < 0.08). We imputed data with regression method (participants were prompted, but not required, to answer any unanswered OSS items; as a result, less than 0.02% of data were missing).

### Results

According to the correlation results (see Table 1A), authentic pride was relatively strongly and positively related to prestige, and weakly to dominance. Furthermore, authentic pride was relatively strongly related to SSS and weakly to OSS. Prestige was relatively strongly and positively related to SSS. This correlation pattern allowed to test whether the link between SSS and authentic pride is mediated by prestige.

**Table 1A:** Correlations between subjective and objective social status, status maintenance strategies and facets of pride.

	Range	Mean (*SD*)	Skewness (*SE*)	Kurtosis (*SE*)	α	1	2	3	4	5	6
1. Authentic pride	1–5	3.46 (0.74)	-0.64 (0.10)	0.43 (0.21)	0.87	—					
2. Hubristic pride	1–4.14	1.86 (0.65)	0.75 (0.10)	0.01 (0.21)	0.84	0.19**	—				
3. Dominance	1–6.30	3.34 (0.84)	0.28 (0.10)	0.24 (0.21)	0.76	0.10**	0.44**	—			
4. Prestige	2.25–6.30	4.37 (0.72)	-0.17 (0.10)	0.01 (0.21)	0.80	0.59**	0.16**	0.24**	—		
5. Subjective social status	1–10	6.56 (1.71)	-0.70 (0.10)	0.19 (0.21)	—	0.60**	0.07	0.10*	0.55**	—	
6. Education^a^	1–6^a^	5.34 (0.96)	-1.5 (0.10)	2.02 (0.21)	—	0.13**	-0.02	-0.05	0.07	0.08*	—
7. Income^b^	1–5^b^	2.60 (1.04)	0.29 (0.10)	-0.51 (0.21)	—	0.19**	-0.04	0.02	0.19**	0.22**	0.30**


**SD*, standard deviation; *SE*, standard error. ^*a*^On a scale of 1 = less than elementary school degree; 2 = finished elementary school; 3 = ongoing high school; 4 = finished high school; 5 = ongoing higher education; 6 = finished higher education. ^*b*^On a scale of 1 = between 0 and 50,000 HUF ∼ 0 and 180 USD; 2 = between 50,001 and 150,000 HUF ∼ 181 and 540 USD; 3 = between 150,001 and 250,000 HUF ∼ 541 and 900 USD; 4 = between 250,001 and 500,000 HUF ∼ 901 and 1,800 USD; 5 = above 500,001 HUF ∼ 1,800 USD. ^∗^*p* < 0.05; ^∗∗^*p* < 0.01.*

On the other hand, hubristic pride was positively related to dominance and was weakly and positively related to prestige, SSS and OSS. This self-reported correlational pattern indicates that OSS plays a minor role in both forms of pride and it is only positively associated with dominance-based status maintenance strategies. Descriptive statistics and inter-factor correlations among the measured variables are presented in Table 1A.

**FIGURE 1:**
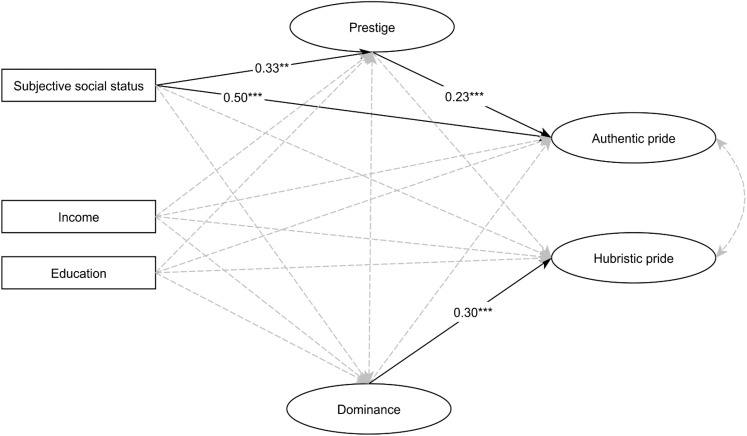
SEM analysis between subjective and objective social status, status maintenance strategies, and facets of pride. Standardized regression weights are presented on the arrows. Dashed line means non-significant relationship. ^∗∗∗^*p* < 0.001.

Figure 1 presents the results of the SEM model (TLI = 0.953, CFI = 0.967, RMSEA = 0.065). Results provided support for the proposed model. Specifically, SSS was indirectly and relatively strongly related to authentic pride via prestige. OSS measures (education and income) were neither related to status maintenance (dominance and prestige) nor facets of pride (authentic and hubristic). Furthermore, dominance was moderately related to hubristic pride. Mediational analysis is presented in Table 1B with statistics on the total, direct and indirect effects with 95% bias-corrected bootstrapped confidence intervals.

**Table 1B:** Study 1: Standardized estimates of total, direct, and indirect effects with 95% bias-corrected bootstrapped confidence intervals.

	Total effect		Direct effect		Indirect effect	
			
	β	95% CI	β	95% CI	β	95% CI
**Subjective social status**						
SSS→Authentic pride	0.573**	[0.507, 0.63]	0.503**	[0.43, 0.57]	0.07**	[0.045, 0.104]
SSS→Hubristic pride	0.089	[0.001, 0.167]	0.069	[-0.029, 0.156]	0.02	[-0.019, 0.065]
**Objective social status**						
Income→Authentic pride	0.037	[-0.034, 0.108]	0.029	[-0.037, 0.099]	0.008	[-0.009, 0.029]
Education→Authentic pride	0.066	[-0.008, 0.140]	0.073	[0.006, 0.149]	-0.007	[-0.026, 0.014]
Income→Hubristic pride	-0.059	[-0.148, 0.038]	-0.062	[-0.149, 0.024]	0.003	[-0.026, 0.033]
Education→Hubristic pride	-0.015	[-0.098, 0.065]	-0.021	[-0.095, 0.058]	0.005	[-0.024, 0.036]


*Bootstrapped confidence intervals were estimated using maximum likelihood. SSS, subjective social status; OSS, objective social status. ^∗∗^*p* < 0.01.*

### Discussion

Study 1 provided initial support for our hypotheses. Results of Study 1 supported that SSS was a more relevant construct regarding pride than OSS which confirmed that pride was the outcome affect of the subjective evaluation of success (Tracy and Robins, 2004) in light of the social group’s feedback on respect. Furthermore, SSS was related to authentic pride via prestige. When individuals perceive respect, admiration and influence in their relevant social groups it will go hand in hand with the usage of prestige-based status maintenance strategies such as sharing knowledge and skills and being helpful with other members of the group. These experiences predispose the individual to feel pride in an authentic way. On the other hand, the usage of dominance-based status maintenance strategies—such as threatening others and being aggressive—can predispose hubristic pride as the result of the arrogant influence on other members of the group.

These findings confirm the evolutionary approach of pride (Cheng et al., 2010). In the case of hubristic pride, when individuals lack prestige based tools to maintain status they will experience that they are conceited, arrogant and pompous. OSS measures had no significant effects neither on status maintenance strategies nor pride. It means that not financial benefits or possessed university degrees were considered as more important symbols regarding what makes one pride but group members’ feedback and evaluation on the given person’s respectedness, admiration, and influence.

In sum, we supposed that SSS and OSS should be taken into consideration independently in status maintenance and pride because they have differentiated effects. On the other hand results can be distorted by not appropriate and less detailed OSS measures. In Study 2, we aimed to overcome this limitation by measuring OSS with multiple related constructs.

## Study 2

In Study 2, we investigated how OSS and SSS are related to status maintenance strategies and pride. Study 2 was a similar self-reported questionnaire study as Study 1, but we aimed to measure OSS with differentiated measures. SEM analysis was carried out to investigate the relationship pattern of OSS and SSS to authentic and hubristic pride with the mediation of status maintenance strategies. The raw data is available on OSF: https://osf.io/ebg8a/.

### Method

#### Participants

A total of 509 Hungarian participants were recruited from topic irrelevant social media groups with more than 10,000 members in the present study (370 females, 135 males, 4 missing), aged between 18 and 75 (*M*_age_ = 27.34 years, *SD*_age_ = 10.26 years). Regarding their place of residence, 249 (48.9%) lived in the capital, 91 (17.9%) lived in county towns, 114 (22.4%) lived in towns, and 52 (10.2%) lived in villages, 3 respondents did not indicate their place of residence. Respondents were also asked about their average monthly income (*M*_Hungarianincome_ = 372 USD as per the Hungarian Central Statistical Office, 2006). The average monthly income for 116 (22.8%) respondents was less than 180 USD, 210 respondents (41.3%) had between 180 and 540 USD, 89 (17.5%) had an average monthly income of 541–904 USD, 64 (12.6%) had 905–1,808 USD monthly income on average, 24 (4.7%) respondents had more than 1,809 USD average monthly income and 6 individuals (1.8%) did not indicate their average monthly income.

#### Measures, Procedure, and Statistical Analysis

In this study, the same scales were used as in Study 1: Hubristic and Authentic Pride Scale (Tracy and Robins, 2007b; α_authentic_ = 0.86, α_hubristic_ = 0.84) in a shortened form. SSS was measured by the MacArthur Scale of Subjective Social Status (Ostrove et al., 2000; Adler and Stewart, 2007).

Objective social status was measured with different status related constructs. Respondents were asked about their average monthly income with the above mentioned categories. Furthermore, financial wealth was asked on a 6-point Likert scale (1 = I live in deliberately good financial circumstances; 2 = I live without financial problems; 3 = I economize but live well; 4 = I almost can live without financial problems; 5 = I have financial problems from month to month; 6 = I live in deprivation). Occupation was coded into two categories, white and blue collar workers. Moreover, respondents were asked about such status related possessions as mobile phone, car, and house. They had to indicate the value of their phone and car on a 10-point scale where 1 indicated the worst and oldest types of phones and cars and 10 indicated the best, latest and most modern phones or cars. It was also illustrated with pictures for better understanding. Respondents were asked about if they live in their own house or not. Regarding the procedure and statistical analysis of this study it was the same as in Study 1. We data with regression method (participants were prompted, but not required, to answer any unanswered OSS items; as a result, less than 0.02% of data were missing).

### Results

According to the correlation results, authentic pride was relatively strongly and positively related to prestige and SSS and weakly and positively to some OSS measures (e.g., income, occupation, and car). Prestige was strongly and positively related to SSS. Hubristic pride was relatively strongly and positively related to dominance. Descriptive statistics and inter-factor correlations among the measured variables are presented in Table 2A.

**Table 2A:** Correlations between subjective and objective social status, status maintenance strategies and facets of pride.

	Range	Mean (*SD*)	Skewness (*SE*)	Kurtosis (*SE*)	α	1	2	3	4	5	6	7	8	9	10
1. Authentic pride	1–5	3.01 (0.91)	-0.08 (0.11)	-0.56 (0.22)	0.89	—									
2. Hubristic pride	1–5	1.61 (0.71)	1.52 (0.11)	2.19 (0.22)	0.84	0.12**	—								
3. Dominance	1–5	2.70 (0.97)	0.39 (0.11)	-0.42 (0.22)	0.75	0.16**	0.41**	—							
4. Prestige	1–5	3.11 (0.83)	-0.13 (0.11)	-0.46 (0.22)	0.70	0.52**	0.23**	0.33**	—						
5. Subjective social status	1–10	6.42 (1.75)	-0.68 (0.11)	0.54 (0.22)	—	0.56**	0.11*	0.22**	0.50**	—					
6. Income^a^	1–5	2.34 (1.11)	0.70 (0.11)	-0.26 (0.22)	—	0.25**	0.07	0.03	0.20**	0.12**	—				
7. Financial wealth	1–7	3.90 (2.35)	0.45 (0.11)	-1.61 (0.22)	—	0.14**	-0.03	-0.04	0.051	0.12*	0.15**	—			
8. Occupation^b^	0, 1	—	—	—	—	0.29**	0.07	-0.13	0.23**	0.20**	0.02	-0.02	—		
9. Phone^c^	1–10	7.33 (1.66)	-0.56 (0.11)	0.17 (0.22)	—	0.18**	0.12*	0.11*	0.19**	0.24**	0.12**	0.10*	-0.02	—	
10. Car^c^	1–10	5.66 (1.76)	0.05 (0.21)	-0.18 (0.42)	—	0.26**	-0.05	0.02	0.28**	0.32**	0.31**	0.16	0.30*	0.30**	—
11. Home^d^	0, 1	—	—	—	—	0.12**	-0.06	-0.07	0.05	0.07	0.02	-0.01	0.12	-0.01	0.02


**SD*, standard deviation; *SE*, standard error. ^*a*^On a scale of 1 = between 0 and 50,000 HUF ∼ 0 and 180 USD; 2 = between 50,001 and 150,000 HUF ∼ 181 and 540 USD; 3 = between 150,001 and 250,000 HUF ∼ 541 and 900 USD; 4 = between 250,001 and 500,000 HUF ∼ 901 and 1,800 USD; 5 = above 500,001 HUF ∼ 1,800 USD. ^*b*^0 indicated blue collar and 1 indicated white collar. ^*c*^On a scale of 1 = the worst and oldest type and 10 the best, the latest and most modern type. ^*d*^0 indicated no possession and 1 indicated possession. ^∗^*p* < 0.05; ^∗∗^*p* < 0.01.*

**FIGURE 2:**
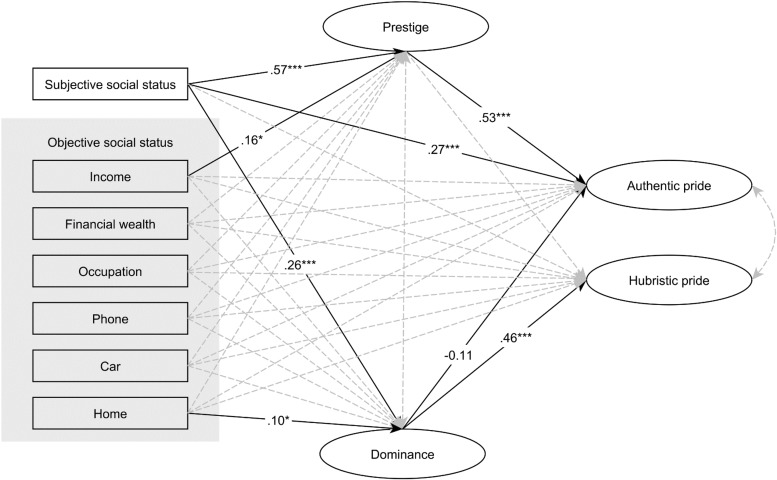
SEM analysis between subjective and objective social status, status maintenance strategies, and facets of pride. Standardized regression weights are presented on the arrows. Dashed line means non-significant relationship. ^∗∗∗^*p* < 0.001.

This correlation pattern allowed to test the hypothesized SEM model. Figure 2 presents the result of SEM analysis (TLI = 0.905, CFI = 0.937, RMSEA = 0.053). Because there were weak correlations among OSS measures, the variables were tested independently in the model, not as an aggregated or latent variable. According to the model, SSS was directly and positively related to prestige and to authentic pride and indirectly to authentic pride via prestige. Furthermore, SSS was directly and moderately related to dominance and to hubristic pride via dominance. Income was negligible weakly related to prestige, and home was negligible weakly related to dominance. All in all, OSS was unrelated to either authentic or hubristic pride and status maintenance strategies as well. Furthermore, dominance was directly and relatively strongly related to hubristic pride. Mediational analysis is presented in Table 2B with statistics on the total, direct, and indirect effects with 95% bias-corrected bootstrapped confidence intervals.

**Table 2B:** Study 2: Standardized estimates of total, direct, and indirect effects with 95% bias-corrected bootstrapped confidence intervals.

	Total effect		Direct effect		Indirect effect	
			
	β	95% CI	β	95% CI	β	95% CI
**Subjective social status**						
SSS→Authentic pride	0.544**	[0.463, 0.614]	0.271**	[0.135, 0.398]	0.273**	[0.182, 0.416]
SSS→Hubristic pride	0.116*	[0.014, 0.219]	-0.068	[-0.196, 0.054]	0.018*	[0.087, 0.288]
**Objective social status**						
Income→Authentic pride	0.102*	[0.004, 0.201]	0.018	[-0.074, 0.11]	0.084	[0.034, 0.155]
Financial wealth→Authentic pride	0.062	[-0.018, 0.142]	0.07	[-0.006, 0.151]	-0.009	[-0.058, 0.038]
Occupation→Authentic pride	0.119*	[0.037, 0.202]	0.122*	[0.047, 0.204]	-0.003	[-0.058, 0.048]
Phone→Authentic pride	0.032	[-0.051, 0.104]	-0.007	[-0.087, 0.062]	0.039	[-0.012, 0.098]
Car→Authentic pride	0.063	[-0.021, 0.145]	0.061	[0–0.029, 0.137]	0.002	[-0.047, 0.054]
Home→Authentic pride	-0.031	[-0.083, 0.014]	-0.047	[-0.120, 0.018]	-0.031	[-0.083, 0.014]
Income→Hubristic pride	0.063	[-0.055, 0.172]	0.03	[-0.083, 0.138]	0.033	[-0.029, 0.098]
Financial wealth→Hubristic pride	-0.067	[-0.153, 0.036]	-0.032	[-0.144, 0.061]	-0.035	[-0.089, 0.014]
Occupation→Hubristic pride	0.008	[-0.097, 0.116]	0.036	[-0.056, 0.137]	-0.028	[-0.085, 0.022]
Phone→Hubristic pride	0.096*	[0.012, 0.178]	0.054	[-0.026, 0.136]	0.042	[-0.003, 0.104]
Car→Hubristic pride	-0.04	[-0.146, 0.062]	-0.044	[-0.141, 0.047]	0.004	[-0.059, 0.063]
Home→Hubristic pride	0.048	[-0.059, 0.141]	0.003	[-0.077, 0.094]	0.045	[-0.008, 0.105]


*Bootstrapped confidence intervals were estimated using maximum likelihood. SSS, subjective social status; OSS, objective social status. ^∗^p < 0.05, ^∗∗^*p* < 0.01.*

### Discussion

Study 2 gave further evidence to the hypothesized relationship pattern. According to the results, SSS was a more relevant construct regarding status maintenance strategies and pride. The relationship between SSS and authentic pride was mediated by prestige which indicates that perceived respect, admiration and influence in relevant social groups go hand in hand with sharing knowledge and skills and make possible to be proud in an authentic way. SSS was also related to hubristic pride with the mediation of dominance. It indicates that SSS can be the source of both sort of status maintenance strategies in which the prestige plays the main role and the dominance has a secondary role. For individuals with high SSS prestige can provide the basis of maintenance of high status, but sometimes it might be relevant or useful to use dominance-based status maintenance strategies, as well. These results can shed light on the proportion of these strategies in which prestige has the main role, but dominance cannot be negligible.

Surprisingly, OSS measures had no significant effects or very small effects on both status maintenance strategies and facets of pride. Suggestion from Study 1, that not possessions and money have to be taken into consideration to feel ourselves proud get evidence. Group members’ subjective evaluation appeared to have a much more important role regarding pride.

Study 2 confirmed that SSS and OSS have to be taken into account differently considering status maintenance strategies and pride. Studies 1 and 2 were self-reported, cross-sectional and correlational studies in which status was not systematically manipulated which can be one of the limitations of these works. Therefore, in Study 3 we intended to manipulate SSS and OSS and investigate their differentiated effects on status maintenance strategies and pride in a situation evaluation task.

## Study 3

In Study 3, our main goal was to investigate systematically how SSS and OSS is related to status maintenance strategies and facets of pride in a vignette study for reducing the potential bias caused by the self-report measure of OSS. For this purpose OSS and SSS were manipulated in a 2 × 2 vignette design. Participants were asked to indicate how proud they would feel and how they would behave to maintain their status. The raw data is available on OSF: https://osf.io/ebg8a/.

### Method

#### Participants

A total of 345 Hungarian participants were recruited from topic irrelevant social media groups with more than 10,000 members in the present study, 69 (20%) of them were dropped out from the analysis because they reported that it was very difficult or rather difficult for them to imagine the described vignette situation. The final sample consisted of 276 participants (222 females, four missing) aged between 18 and 70 (*M*_age_ = 28.78 years, *SD*_age_ = 11.99 years). Regarding their place of residence, 109 (39.5%) lived in the capital, 33 (12.0%) lived in county towns, 95 (34.4%) lived in towns, and 35 (12.7%) lived in villages, 4 respondents did not indicate their place of residence.

Respondents were asked about their financial wealth. Sixty-six participants (23.9%) reported that s/he lives in without financial problems, 134 (48.6%) reported that s/he economizes but live well, 37 (13.4%) reported that s/he can almost live without financial problems, 13 (4.7%) reported that s/he has financial problems from month to month, five participants (1.8%) reported that s/he lives in deprivation and 17 respondents did not answer this question.

#### Measures and Procedure and Statistical Analysis

A vignette study was carried out to investigate the relationship pattern between the two forms of social status, status maintenance strategies, and facets of pride. SSS and OSS were manipulated (high or low) in a 2 × 2 design across the vignettes. First, respondents were asked to imagine that they are in the situation characterized by the vignette. They were instructed to imagine that they hold a presentation at a company and report their success which was 20% higher than the expected key performance indicators. OSS was manipulated along level of education, financial situation, phone, type of home, and clothes. *High objective social status* was characterized by a degree from a university with high reputation, having the latest iPhone, fashionable clothes, an own flat and living without financial problems. *Low objective social* status was characterized by having vocational school degree, low-end cellphone, non-fashionable clothes, renting a small flat with acquaintances, and having some financial problems. In *high subjective social status* conditions the respondents had to imagine that they were admired and respected by colleagues and in *low subjective social status* conditions they were not admired and respected by colleagues. Appendix contains the full text of the vignettes.

The research was performed with an online questionnaire system and participants were randomly assigned into one of four conditions. First, they were informed about the goals and the content of the study. All subjects gave written informed consent in accordance with the Declaration of Helsinki. They were also assured the anonymity of their answers. Afterward participants were asked to answer a three-item version of the Dominance and Prestige Scale (Cheng et al., 2010; α_prestige_ = 0.78, α_dominance_ = 0.55) and a shortened version of the Hubristic and Authentic Pride Scale (Tracy and Robins, 2007b; α_authentic_ = 0.91, α_hubristic_ = 0.87). Finally, participants responded to demographic questions.

Regarding the statistical analysis of this study it was the same as in Studies 1 and 2 except for the conditions in dummy variables. Regarding both OSS and SSS low levels were coded as 0, and high levels were coded as 1.

### Results

Descriptive statistics of the measured scales in the four conditions (OSS – high/low, SSS – high/low) are presented in Table 3. Both pride measures had the highest scores when both OSS and SSS were high. Prestige were higher when OSS was low and SSS was high (compared to high OSS-low SSS and low OSS-low SSS). Dominance were higher when OSS was high and SSS was low (compared to low OSS-high SSS and low OSS-low SSS). These results implicate that high SSS is more relevant regarding authentic pride and prestige and OSS is more relevant regarding hubristic pride and dominance.

**Table 3A:** Descriptive statistics by groups for authentic pride, hubristic pride, prestige, and dominance.

		Scale	Range	Mean	*SD*
High objective social status	High subjective social status	a. Authentic pride	2.40–5	4.09	0.67
		b. Hubristic pride	1–5	1.69	0.81
		c. Prestige	1–5	3.01	0.82
		d. Dominance	1.33–5	2.57	0.78
	Low subjective social status	e. Authentic pride	1.60–5	3.69	0.89
		f. Hubristic pride	1–3.80	1.65	0.73
		g. Prestige	1–4.7	2.79	0.82
		h. Dominance	1.33–5	2.68	0.83
Low objective social status	High subjective social status	i. Authentic pride	1–5	3.56	0.89
		j. Hubristic pride	1–2.60	1.23	0.36
		k. Prestige	1–4.7	3.07	0.81
		l. Dominance	1.33–4.7	2.48	0.80
	Low subjective social status	m. Authentic pride	1–5	2.82	0.99
		n. Hubristic pride	1.3	1.18	0.39
		o. Prestige	1–5	2.48	0.95
		p. Dominance	1.33–4.33	2.48	0.80


*All variables were measured on a 5-point Likert scale.*

Two-way ANOVA was conducted to compare the main effects of SSS and OSS and the interaction effect between SSS and OSS on authentic and hubristic pride. Regarding authentic pride, SSS had a significant main effect [*F*(1,272) = 28.56, *p* < 0.001], indicating a significant difference between low SSS (*M*_low_ = 3.27, *SD*_low_ = 1.05) and high SSS conditions (*M*_high_ = 3.80, *SD*_high_ = 0.84). OSS had a significant main effect on authentic pride as well [*F*(1,272) = 44.31, *p* < 0.001], indicating a significant difference between low OSS (*M*_low_ = 3.21, *SD*_low_ = 1.01) and high OSS conditions (*M*_high_ = 3.88, *SD*_high_ = 0.82). The interaction effect was not significant [*F*(1,272) = 2.52, *p* = 0.113]. Regarding hubristic pride SSS did not have a significant main effect [*F*(1,272) = 0.418, *p* = 0.518]. In contrast, OSS has a significant main effect on hubristic pride [*F*(1,272) = 41.60, *p* < 0.001], indicating a significant difference between low OSS (*M*_low_ = 1.21, *SD*_low_ = 0.35) and high OSS conditions (*M*_high_ = 1.70, *SD*_high_ = 0.76). The interaction effect was not significant [*F*(1,272) = 0.002, *p* = 0.967].

**FIGURE 3:**
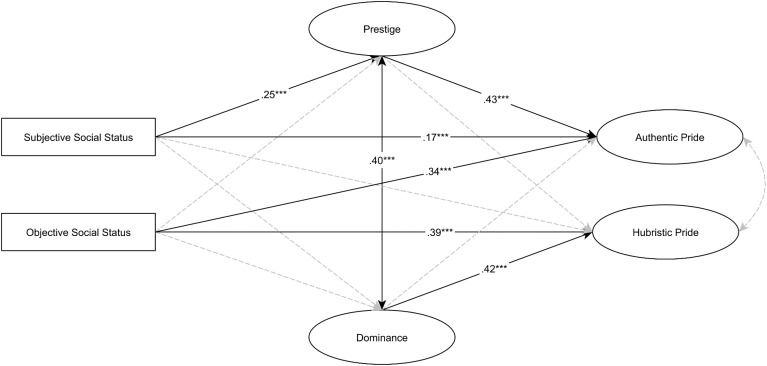
SEM analysis between subjective and objective social status, status maintenance strategies, and facets of pride. Standardized regression weights are presented on the arrows. Dashed line means non-significant relationship. ^∗∗∗^*p* < 0.001. Levels of SSS and OSS are coded as 0-low, 1-high.

Figure 3 presents the results of the SEM analysis (TLI = 0.946, CFI = 0.957, RMSEA = 0.055). Mediational analysis is presented in Table 3B with statistics on the total, direct, and indirect effects with 95% bias-corrected bootstrapped confidence intervals. SSS was directly and positively related to prestige and indirectly and positively to authentic pride via prestige. OSS was directly related to authentic and hubristic pride. Dominance was related to hubristic pride.

**Table 3B:** Study 3: Standardized estimates of total, direct, and indirect effects with 95% bias-corrected bootstrapped confidence intervals.

	Total effect		Direct effect		Indirect effect	
			
	β	95% CI	β	95% CI	β	95% CI
**Subjective social status**						
SSS→Authentic pride	0.288^∗∗^	[0.182, 0.393]	0.171^∗∗^	[0.057, 0.286]	0.117 **	[0.055, 0.206]
SSS→Hubristic pride	0.044	[-0.083, 0.156]	0.052	[-0.061, 0.156]	-0.008	[-0.09, 0.069]
**Objective social status**						
OSS→Authentic pride	0.369^∗∗^	[0.276, 0.479]	0.343^∗∗^	[0.251, 0.453]	0.026	[-0.03, 0.087]
OSS→Hubristic pride	0.386^∗∗^	[0.291, 0.483]	0.338^∗∗^	[0.239, 0.443]	0.048	[-0.015, 0.115]


*Bootstrapped confidence intervals were estimated using maximum likelihood. SSS, subjective social status; OSS, objective social status. ^∗^*p* < 0.05; ^∗∗^*p* < 0.01.*

### Discussion

Study 3 gave new aspects to the hypothesized relationship pattern. SSS was related to authentic pride via prestige as in Studies 1 and 2. Moreover, in Study 3 also OSS had significant relationship to authentic and to hubristic pride as well. Regarding authentic pride it means that if the individual perceives (1) respect, admiration and influence in relevant social groups and also (2) has money, lives well, possesses a good phone and own home enables to feel accomplishment and confidence. On the other hand, in this imagined situation SSS was not related to hubristic pride. It means that when individuals imagine themselves as having a lot of money and possessions but others do not respect them they report that they would feel arrogant and conceited. Dominance group means in high OSS-high SSS and high OSS-low SSS confirm this.

Study 3 also confirmed that SSS and OSS have different effect on status maintenance strategies and pride as well. Although Study 3 was also a self-report measure which allows positive self-serving bias. It may bias results for the following reasons: (1) individuals do not tend to confess that they are dominant or hubristic (low group means may confirm this statement) and (2) financial questions might be seen too intimate which can undermine honest answers. For this reason, in Study 4 we asked participants to rate a hypothetical other person in the same situation in order to avoid these negative effects of self-serving bias.

## Study 4

In this vignette study, our main goal was reducing self-serving biases in the assessment of the relationship pattern of SSS, OSS, prestige, dominance, authentic and hubristic pride within a vignette study highly similar to the previous one. The only difference was related to the perspective of responding. In the previous study, participants imagined themselves in the role of the successful person, in the present case they were requested to evaluate someone else’s emotions and supposed behavior. For this purpose, we used the 2 × 2 research design of Study 2 in which SSS and OSS were manipulated with this only one modification. The raw data is available on OSF: https://osf.io/ebg8a/.

### Method

#### Participants

A total of 497 Hungarian participants were recruited from topic irrelevant social media groups with more than 10,000 members in the present study (379 females), aged between 18 and 64 (*M*_age_ = 28.25 years, *SD*_age_ = 9.19 years). Regarding their financial situation 128 respondents (25.8%) indicated that he/she lives without financial problems, 229 respondents (46.1%) indicated that he/she economize but live well, 88 respondents (17.7%) indicated that he/she almost can live without financial problems, 23 respondents (4.6%) indicated that he/she has financial problems from month to month, 5 respondents (0.1%) indicated that lives with deprivation and 24 respondents (4.8%) had given no answer.

#### Measures and Procedure and Statistical Analysis

A vignette study was carried out to investigate the relationship pattern between the two forms of social status, status maintenance strategies, and facets of pride. SSS and OSS were manipulated (high or low) in a 2 × 2 design across the vignettes. The storyline was the same as in Study 3, but in the present study participants were requested to evaluate someone else’s emotions and supposed behavior. Respondents read a short story about “Gabi” (which is a gender-neutral name in Hungarian). Gabi’s OSS and SSS were manipulated in a same way as in Study 3. Regarding the procedure and statistical analysis of this study, it was the same as in Study 3 (α_prestige_ = 0.88, α_dominance_ = 0.77, α_authentic_ = 0.84, α_hubristic_ = 0.91).

### Results

Descriptive statistics of the measured scales in the four conditions (OSS – high/low, SSS – high/low) are presented in Table 4A. Prestige scores were higher than dominance scores when SSS was high regardless of the level of OSS. Consequently dominance scores were higher than prestige scores when SSS was low regardless of the level of OSS.

**Table 4A:** Descriptive statistics by groups for authentic pride, hubristic pride, prestige, and dominance.

		Scale	Range	Mean	*SD*
High objective social status	High subjective social status	a. Authentic pride	1–5	4.30	0.85
		b. Hubristic pride	1–5	2.11	0.96
		c. Prestige	1.33–5	3.41	0.82
		d. Dominance	1–3.67	2.54	0.65
	Low subjective social status	e. Authentic pride	1–5	4.18	0.82
		f. Hubristic pride	1–5	1.19	0.39
		g. Prestige	1–4.67	1.71	0.74
		h. Dominance	1–4.33	2.66	0.76
Low objective social status	High subjective social status	i. Authentic pride	1.67–5	3.80	0.91
		j. Hubristic pride	1–3.33	1.19	0.39
		k. Prestige	1–5	3.50	0.85
		l. Dominance	1–4	2.12	0.55
	Low subjective social status	m. Authentic pride	1.33–5	3.42	0.91
		n. Hubristic pride	1–3.33	1.24	0.51
		o. Prestige	1–4.33	1.79	0.80
		p. Dominance	1–3.67	2.19	0.58


*All variables were measured on a 5-point Likert scale.*

**Table 4B:** Study 4: Standardized estimates of total, direct, and indirect effects with 95% bias-corrected bootstrapped confidence intervals.

	Total effect		Direct effect		Indirect effect	
			
	β	95% CI	β	95% CI	β	95% CI
**Subjective social status**						
SSS→Authentic pride	0.147**	[0.065, 0.241]	-0.156	[-0.325, 0.035]	0.303**	[0.153, 0.452]
SSS→Hubristic pride	-0.112*	[-0.182, -0.032]	0.014	[-0.104, 0.153]	-0.126	[-0.262, -0.013]
**Objective social status**						
OSS→Authentic pride	0.393**	[0.308, 0.479]	0.317**	[0.213, 0.431]	0.077*	[0.005, 0.148]
OSS→Hubristic pride	0.57**	[0.517, 0.627]	0.245**	[0.161, 0.332]	0.325**	[0.257, 0.401]


*Bootstrapped confidence intervals were estimated using maximum likelihood. SSS, subjective social status; OSS, objective social status. ^∗^*p* < 0.05; ^∗∗^*p* < 0.01.*

Two-way ANOVA was conducted to compare the main effects of SSS and OSS and the interaction effect between SSS and OSS on authentic and hubristic pride. Regarding authentic pride, SSS had a significant main effect [*F*(1,493) = 10.35, *p* < 0.01], indicating a significant difference between low SSS (*M*_low_ = 3.80, *SD*_low_ = 0.94) and high SSS conditions (*M*_high_ = 4.05, *SD*_high_ = 0.91). OSS had a significant main effect on authentic pride as well [*F*(1,493) = 64.67, *p* < 0.001], indicating a significant difference between low OSS (*M*_low_ = 3.62, *SD*_low_ = 0.92) and high OSS conditions (*M*_high_ = 4.24, *SD*_high_ = 0.83). The interaction effect was not significant [*F*(1,493) = 2.88, *p* = 0.09]. Regarding hubristic pride SSS had a significant main effect [*F*(1,493) = 7.81, *p* < 0.01], indicating a significant difference between low SSS (*M*_low_ = 1.85, *SD*_low_ = 1.04) and high SSS conditions (*M*_high_ = 1.65, *SD*_high_ = 0.86), but this difference was very small. OSS also had a significant main effect on hubristic pride [*F*(1,493) = 216.15, *p* < 0.001], indicating a significant difference between low OSS (*M*_low_ = 1.23, *SD*_low_ = 0.45) and high OSS conditions (*M*_high_ = 2.27, *SD*_high_ = 1.04). The interaction effect was not significant [*F*(1,493) = 3.58, *p* = 0.06].

**FIGURE 4:**
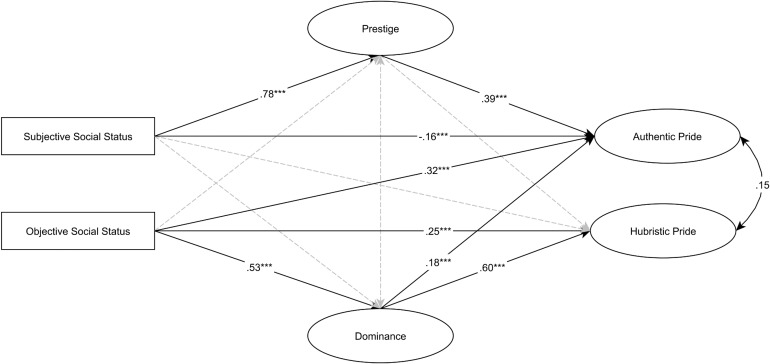
SEM analysis between subjective and objective social status, status maintenance strategies, and facets of pride. Standardized regression weights are presented on the arrows. Dashed line means non-significant relationship. ^∗∗∗^*p* < 0.001. Levels of SSS and OSS are coded as 0-low, 1-high.

Figure 4 presents the results of the SEM analysis (TLI = 0.953, CFI = 0.967, RMSEA = 0.065). Mediational analysis is presented in Table 4B with statistics on the total, direct and indirect effects with 95% bias-corrected bootstrapped confidence intervals. SSS was indirectly and strongly related to authentic pride via prestige. SSS was also directly related to authentic pride with a small but negative regression weight which is caused by a suppression effect, when the indirect effect is so strong that it overwhelms the direct effect (MacKinnon et al., 2000; Paulhus et al., 2004). Sobel test was used to evaluate the significance of suppressor effect (*zs* = 4.49, *p* < 0.0001). SSS was indirectly related to hubristic pride via prestige but with a negligible small negative regression weight.

Objective social status was directly related to authentic pride and to hubristic pride. OSS was also indirectly related to hubristic pride via dominance. This mediational relationship pattern was justified only in this study and coefficients were relatively strong, furthermore an unexpected positive association emerged between dominance and authentic pride but with a small coefficient.

### Discussion

Study 4 provided new insights into the relationship pattern of the different aspects of status, status maintenance, and pride. SSS was related to authentic pride via prestige as in Studies 1, 2, and 3. OSS was directly related to both authentic and hubristic pride as in Study 3, but in Study 4 OSS was indirectly and strongly related to hubristic pride via dominance. It means that in the evaluation of another person, participants tend to use different aspects of status in contrast to when they are requested to evaluate themselves. It appears that in other’s evaluation different manifestations of OSS can get more emphasis regarding status maintenance and pride. It is especially true regarding the links between OSS → dominance → hubristic pride path. With other words, when this imagined person had high OSS (lot of money and possessions) with low SSS (lack of respect and admiration from the relevant social group members) this person was perceived to use dominant status maintenance strategies and to experience hubristic pride.

We suppose that the stronger presence of OSS can be related to the reduced effect of self-serving biases. Furthermore, in the present experimental manipulation, participants could rely their decisions on visible cues that they can see on other persons (material goods, quality of cellphone, clothes) that people use for social categorization frequently but which might be more unnoticed if individuals evaluate themselves. In the latter case, one might put more emphasis on the internal experiences, feelings and thoughts that are just partly accessible in the case of other persons. These results will be further detailed in the general discussion in light of the results of Study 3.

In sum, Study 4 also confirmed that it is worth to separate the effects of SSS and OSS regarding prestige, dominance, authentic and hubristic pride. Furthermore, this study provided empirical evidence to a new perspective in pride research with changing the evaluative perspective which can reduce self-serving biases and provide a detailed picture on the OSS, dominance and hubristic pride.

## General Discussion

Pride is a status-related emotion. However, the nature of status appears to be an understudied phenomenon in pride research. In the present research project, four studies provided evidence for the differentiated role of SSS and OSS in status maintenance strategies and pride. Our main result was that SSS—in contrast to OSS—was more strongly related to authentic pride via prestige. Regarding the role of OSS in status maintenance strategies and pride it had different effects depending on the design of the study. In the questionnaire studies (Studies 1 and 2) OSS was unrelated to both status maintenance and facets of pride. However, in the vignette studies (Studies 3 and 4) when participants had to evaluate a stereotypical situation OSS played a more significant role in facets of pride. Despite these general tendencies, the four studies could provide a more differentiated picture about the relationship pattern between social status, status maintenance strategies and facets of pride and pride cannot be dealt as a homogenous construct in pride research.

### The Role of Subjective Social Status in Authentic Pride

All four studies have confirmed that contrasting to OSS, SSS has a more central role in pride. This result indicates that pride is the outcome of a personal subjective evaluation of status that reflects rather the sum of social feedback received from group members than such objective measures as education, goods or wealth. In all four studies SSS was related to authentic pride via prestige. These results indicate that individuals, who are appreciated by friends, family, and colleagues (high SSS), often share their knowledge, skills and are helpful (prestige), while experiencing accomplishment, confidence and success (authentic pride). This whole cycle can be explained by the Matthew-effect (Merton, 1968) postulating the “rich get richer” principle in which a positive feedback loop can be generated regarding social feedback (Petersen et al., 2011; de Rijt et al., 2014). Individuals with higher ranks on the subjective social ladder tend to use socially accepted prestige-based status maintenance strategies and experience the authentic pride which is also socially accepted (Williams and DeSteno, 2009). For this reason it is not surprising that they become socially more accepted and appreciated, which in turn, can result in a positive feedback circle.

### The Apparently Missing Link Between Objective Social Status, Dominance, Prestige, and Facets of Pride

In the first two questionnaire studies OSS either played a non-significant or a negligible role in status maintenance strategies and facets of pride. These results indicate that when individuals evaluate themselves (reporting about the self), objective status (income, goods, or education) is not associated to status maintenance strategies and pride. However, when manipulating OSS systematically (Study 3), it had an effect on both authentic and hubristic pride, but it was still unrelated to status maintenance strategies. In the situation evaluation task (Study 4), in which another person was evaluated instead of the self, OSS was associated with dominance. There are more possible explanations of these apparently missing links of OSS.

One explanation could be that individuals tend to use different signals of status when they observe other people and make opinions about their behavior and emotions in contrast to when they observe their own behavior and inner states. When evaluating others (vs. the self), visual cues of status (e.g., clothes, car or cellphone) might become more important signals. The higher salience of these cues of OSS can make individuals to draw conclusions about how others maintain status or how proud they might be. It can be an example of the correspondence bias (Jones and Davis, 1965; Pronin et al., 2004), when individuals tend to draw inferences from observed behavior to one’s dispositions. This might be the reason of the relatively strong association between OSS (e.g., observed visible characteristics) and dominance status maintenance strategies (e.g., aggression-related dispositions) if others are evaluated (see Study 4). However, this link is missing (see Study 3), when participants report about their own aggressive status maintenance strategies, which can be mainly attributed to situational factors (based on the actor–observer asymmetry of Jones and Nisbett, 1971). In sum, these results suggest that evaluating the self vs. others can have serious implications regarding the relationship pattern of social status, its maintenance and pride.

The missing link between OSS and other constructs in the questionnaire studies can derive from methodological considerations regarding the signals of objective status. In Study 1, it was characterized by only two dimensions (level of education in six main categories and income in five categories). In order to obtain more detailed OSS data, in Study 2, we assessed additional OSS indicators in terms of financial wealth and material possessions, but no relevant links of OSS were found. Furthermore, the intercorrelations between OSS indicators were not strong (see Table 2A). Therefore, we cannot claim that there were participants with unequivocally high OSS and unequivocally low OSS. Consequently, in the following vignette studies, instead of more and more precise assessments, we manipulated OSS in a stereotypical way and shifted all indicators to a high level or low level. In sum, despite our efforts to identify appropriate self-reported measures of OSS, the assessment of OSS is a complicated issue in which obtaining “objective” OSS data (i.e., pay check, material goods, debts, etc.) can be the next step in future studies.

There were other inconsistencies in the present research. Despite our expectations, in the vignette studies (Studies 3 and 4), we found that OSS was related to *both* authentic and hubristic pride. This inconsistency can be explained on the basis of the theory of locus of control (Rotter, 1966). Internal locus of control means that success derives from efforts, abilities or behaviors, whereas in the case of external locus, external factors such as fate, luck or nepotism are responsible for the success. According to the definition of authentic pride, it is attributed to internal, unstable causes while hubristic pride is attributed to internal, stable causes (Tracy and Robins, 2007b). We suppose that OSS is related to authentic pride if the “objective” success is attributed to efforts or abilities (internal locus), while OSS is related to hubristic pride if the “objective” status attributed to external causes (external locus). Further research is required to explore the potential role of locus of control in these relationship patterns. It is possible that experiencing control over success can be related to prestige as status maintenance, while the lack of control can lead to more desperate strategies such as dominance.

All in all, in the case of the questionnaire studies (Study 1 and Study 2) it appears that the OSS did not play any role in pride and status maintenance strategies, while SSS shows a consistent relationship pattern. In these assessment situations participants wrote about their own situation, and perception regarding their status, its maintenance and pride. However, in the case of vignette studies (Study 3 and Study 4) they report their opinion about imagined situations in which they can observe themselves and other people from an idealistic perspective in which all objective status indices are in line (high education, own apartment, high end goods, high income, etc.). As in Hungary as a materialist country (Hofmeister and Neulinger, 2013), the combination of these idealistic material possessions can be related to well-being. In the current circumstances of the respondents there is no link between OSS and authentic pride, but it is possible that in the imagined, idealistic situation respondents may believe that the possession of these things can lead to a certain satisfaction that appears in the form of authentic pride (accomplishment, success, etc.).

### Applied Implications

This research has a few practical implications. On the basis of the results, if the goal is to enhance the subjective experience of authentic pride—in terms of making co-workers or students feel accomplished, successful and fulfilled—it might be advisable to promote SSS rather than OSS incentives. For example, in workplace or school situations, it might be advised to establish and reinforce mutual respect among co-workers and put emphasis on norms promoting effort-based mutual appreciation that can be the background climate for hierarchy formation. However, objective status-based incentives do not appear to have similarly adaptive effect in terms of status maintenance or the subjective experience of pride.

### Limitations and Future Studies

Although the present research aimed to be pioneer investigating the differentiated role of social status in pride, it is not without its limitations. First, female respondents were over-represented in the samples and the samples were not representative. Future studies should implement more balanced and comprehensive samples. Second, no behavioral measures were used. Third, in Study 1 and Study 2 social desirability, in Study 4 availability bias could distort the results. Future studies should use not only self-report and situation evaluation task but make more effort to reduce or eliminate these biases. Furthermore, Study 3 was a situation evaluation task with an imaginary scenario in which respondents were requested to indicate how they would behave and feel in that situation which can be dissimilar to their real-life reactions. Moreover, Study 4 can also provide only limited information about how respondents would evaluate someone with similar behavior in real-life situation.

Another limitation of this research comes from the broad definition of SSS. According to the original instruction of the MacArthur ladder by Adler et al. (2000), participants can think of different social groups when they evaluate their positions. Based on Adler et al.’s (2000) research, it is known that most people define community as their neighborhood (57%), city or town (37%), religious groups (22%), social supporters (20%), workplace (18%), family (18%), friends (12%), people who share their interests (12%), their region (12%), and, finally the nation or world (10%). In Studies 3 and 4, high and low SSS was presented in a workplace environment. Due to this, the social group chosen by the participant may not be irrelevant to OSS. The precise conceptualization of the content of SSS and its relationship to OSS regarding domain-specific status-maintenance strategies and pride may be an important area for future research. Furthermore, previous studies showed that OSS is related to SSS (e.g., Kim et al., 2017), which can also distort the results. However, in Studies 1 and 2, significant but relatively weak correlations were found between OSS indicators and SSS.

Future studies should aim to reduce these above mentioned biases for example with experimental designs. Vignette method is a bridge between questionnaires and experiments and appeared to be a good path to follow. Furthermore, as mentioned above, the relationship between SSS and OSS is a bit unclear, because it may depend on the reference group for SSS. To precise the content of SSS and its relationship with OSS, can be a fruitful area regarding the social dynamics and appraisal processes of pride or maybe investigating domain-specific status-maintenance strategies and domain-specific pride. Furthermore, to get deeper understanding of this relationship pattern, additional constructs can be taken into consideration. Based on previous studies (Lange and Crusius, 2015; Crusius and Lange, 2017), envy can be one of them especially in those situations when others’ evaluation is the goal. In addition, to draw causal conclusions longitudinal studies should be carried out, investigating how changes in OSS and SSS changes over time can influence status maintenance strategies and the two facets of pride. It can be especially true, if one examines status-relevant transition periods, for example before and after (deserved and undeserved) promotions.

## Conclusion

Despite pride is a status-related self-conscious emotion, surprisingly little is known about the differentiated effect of different aspects of status on this emotion. The present study aimed to identify the relationship pattern between status maintenance strategies, the two facets of pride and the two most basic form of status: its subjective and objective aspects. The questionnaire and vignette results showed a few consistent results. One of these is the link between subjective status, prestige maintenance strategies and authentic pride. However, beside this statement, the present research opens more questions that it can answer. According to the type assessment method (self-reported questionnaires vs. evaluation of a hypothetical scenario) and according to the evaluative perspective (self-relevant or other-relevant), the link between objective status with strategies and pride can be different. For these reasons, we can confidently claim that pride is a subjective status-related emotion, but we should be more uncertain to claim that it is an objective status-related one.

## Author Contributions

HB and GO conceived and designed the studies. HB performed the studies. HB, BB, and IT-K analyzed the data. HB, BB, IT-K, and GO wrote the paper.

## Conflict of Interest Statement

The authors declare that the research was conducted in the absence of any commercial or financial relationships that could be construed as a potential conflict of interest.

## APPENDIX: Study 3 vignettes

### OSS Low–SSS Low

Imagine that you are 25 years old, have a vocational school degree, and have been working for a telecommunicational multinational company for 2 years. You make both ends meet although not spend too much money. You don’t have money for the latest phone or expensive clothes. You live in a flat where you rent one room and one of your acquaintances rent the other one.

You give a presentation at your company to your boss in which you present your success, which was 20% higher than the expected key performance indicators. Your boss appreciates your work and reward you with a new laptop.

Your colleagues do not really respect and admire you and your word doesn’t count for them.

### OSS Low–SSS High

Imagine that you are 25 years old, have a vocational school degree, and have been working for a telecommunicational multinational company for 2 years. You make both ends meet although not spend too much money. You don’t have money for the latest phone or expensive clothes. You live in a flat where you rent one room and one of your acquaintances rent the other one.

You give a presentation at your company to your boss in which you present your success, which was 20% higher than the expected key performance indicators. Your boss appreciates your work and reward you with a new laptop.

Your colleagues do respect and admire you and your word count for them.

### OSS High–SSS Low

Imagine that you are 25 years old, have a university degree from Corvinus [a university with high reputation in Hungary] have been working for a telecommunicational multinational company for 2 years. You have the latest iPhone, fashionable clothes and live in your own flat without financial problems.

You give a presentation at your company to your boss in which you present your success which was 20% higher than the expected key performance indicators. Your boss appreciates your work and reward you with a new laptop.

Your colleagues do not really respect and admire you and your word doesn’t count for them.

### OSS High–SSS High

Imagine that you are 25 years old, have a university degree from Corvinus [a university with high reputation in Hungary] have been working for a telecommunicational multinational company for 2 years. You have the latest iPhone, fashionable clothes and live in your own flat without financial problems.

You give a presentation at your company to your boss in which you present your success which was 20% higher than the expected key performance indicators. Your boss appreciates your work and reward you with a new laptop.

Your colleagues do respect and admire you and your word count for them.
